# The clinicopathological significance of Ki67 in papillary thyroid carcinoma: a suitable indicator?

**DOI:** 10.1186/s12957-018-1384-8

**Published:** 2018-05-31

**Authors:** Jingdong Tang, Chunyi Gui, Shenglong Qiu, Min Wang

**Affiliations:** 1 Department of General Surgery, Shanghai General Hospital, Shanghai Jiaotong University School of Medicine, No. 100 Haining Road, Shanghai, 200080 China; 2 Nursing Department, Eye & ENT Hospital, Fudan University, No. 83 Fenyang Road, Shanghai, 200031 China; 3 Department of Surgery, Shanghai Pudong Hospital, Fudan University, No. 2800 Gongwei Road, Shanghai, 201399 China; 4 Oncology Department, Johns Hopkins Hospital, 1800 Orleans Street, Baltimore, MD 21287 USA

**Keywords:** Papillary thyroid carcinoma, Ki67, Thyroiditis

## Abstract

**Background:**

To explore Ki67 expression in papillary thyroid carcinoma (PTC) and its clinical-pathological significance.

**Methods:**

A total of 776 consecutive PTC and benign thyroid disease patients underwent thyroidectomy at Shanghai General Hospital from January 2013 to December 2015 and were retrospectively analysed. Ki67 expression was determined in the PTC and benign thyroid disease tissues, and other clinicopathological factors were identified via statistical analyses.

**Results:**

The Ki67 expression intensity in the PTC group was significantly higher than that in the benign thyroid disease group. In the PTC group, a tumour size ≥ 1 cm and coexistence with thyroiditis were significantly associated with the Ki67 expression intensity. The TGAb and TPOAb plasma levels were linearly correlated with the Ki67 expression intensity. Moreover, the tumour size and Ki67 expression intensity also showed a linear correlation. Receiver operating characteristic (ROC) curve analysis suggested that the optimal cut-off value of Ki67 was 2.50%. Two groups divided by Ki67 cut-off values showed significant differences in the recurrence survival rate.

**Conclusions:**

Ki67 is a suitable biomarker for distinguishing PTC from benign thyroid disease. Ki67 expression was related to the tumour size, thyroiditis and plasma levels of TGAb and TPOAb in PTC. Ki67 could be used as a prognostic indicator in PTC. Patients with high Ki67 expression are more likely to experience disease recurrence.

## Background

Ki67 expression is closely related to cell proliferation and growth. Currently, Ki67 has become one of the most commonly used biomarkers for assessing cell proliferation. Ki67 is generally used as a prognostic indicator and as a tool for diagnostic and research purposes. Many publications have reported high expression of Ki67 in several types of carcinoma [1–3]. Many studies investigating the Ki67 expression and clinical outcomes have been published within the last decade. Most studies of Ki67 expression as it is related to clinical outcomes are concerned with its prognostic predictive value [4–8]. For example, some studies have demonstrated that Ki67 has predictive value in prostate cancer [9, 10] such that a certain case can be treated in an individualized manner based on Ki67 expression. In breast cancer, measuring the expression intensity of Ki67 expression by immunohistochemistry staining is a routine approach for prognostic evaluation [11]. In addition, the St. Gallen Expert Panel 2011 claimed that Ki67 scores ≥ 14% could be used to distinguished molecular subtype luminal B from luminal A disease [12].

The incidence of thyroid carcinoma has increased continuously and dramatically since the 1990s [13, 14]. Currently, thyroid carcinoma is the 5th most common cancer among women; however, it was ranked in the 14th position two decades ago. The epidemiological investigation showed that the increase thyroid carcinoma incidence was largely due to papillary thyroid carcinoma (PTC). PTC is the most common type of thyroid carcinoma, accounting for 80–85% of all thyroid carcinomas [15, 16]. Although most PTCs present indolent features, some patients with aggressive tumours still have poor prognoses [17, 18]. The roles of Ki67 in PTC and thyroid disease still remain unclear. In the present study, we further explored the impact of Ki67 on diagnostic and prognostic values in PTC and thyroid disease.

## Methods

Between January 2013 and December 2015, a total of 799 consecutive PTC and benign thyroid disease patients underwent thyroidectomy at Shanghai General Hospital and were enrolled in the current study. The benign thyroid disease cases included thyroid adenomas and thyroid nodular goiters. There were 571 cases in the PTC group and 228 cases in the benign group.

The PTC group contained 571 cases, including 137 males and 434 females. The average age was 47 years (47.13 ± 13.08 years), and the average tumour size was 1.07 cm (1.07 ± 0.86 cm). In our hospital, central lymph node dissection (CLND) was routinely performed for PTC patients. Patients with unilateral tumours (*n* = 482) underwent thyroid lobectomy and isthmusectomy plus ipsilateral CLND. Patients with bilateral tumours (*n* = 89) underwent total thyroidectomy plus bilateral CLND. The parathyroid glands and recurrent laryngeal nerve were carefully reserved during the surgery. All specimens were examined by at least two experienced pathologists, and the pathology parameters, such as tumour multifocality, extrathyroidal extension and lymph node metastasis (LNM), were recorded.

Respectively, there were 228 cases (including 180 nodular goiter and 48 thyroid adenoma cases) with an average age of 52 years (52.24 ± 12.53), including 57 males and 171 females, in the benign thyroid disease specimen group.

In this study, tumours were considered multifocal when two or more tumour foci were observed in the same or different lobes of the gland. Tumour size was considered to be the predominant nodule diameter. The clinical-pathological staging was based on the 2014 NCCN guidelines. The following parameters were collected: age, gender, coexisting thyroiditis, lymph node metastasis, tumour size, multifocality, extrathyroidal extension and blood biochemical measurements of pre-operation plasma levels of thyroglobulin antibody (TGAb), thyroid peroxidase antibody (TPOAb) and thyroid stimulating hormone (TSH).

Ki67 expression was determined by immune staining with monoclonal antibodies. Monoclonal antibodies for Ki67 were purchased from Gene Tech Company Limited, Shanghai, China. Paraffin-embedded tissue samples were treated with xylene at 48 °C for 3 h to remove paraffin and then washed in distilled water. The samples were then washed in PBS, and antigen retrieval was conducted by treating the samples with pH 6.0 sodium citrate for 5 min in a microwave. The samples were again washed in PBS and treated with 3% hydrogen peroxide for 30 min. Afterwards, the samples were incubated with a Ki67 primary antibody (1:300 dilution) at 4 °C for 20 h. The specimens were then washed in PBS again and incubated with secondary antibodies for 2 h at room temperature. The biotin-peroxidase complex was applied for 1 h and then removed by washing. Finally, the slides were coloured with DAB for 15 min. When the staining was clearly observed in the nucleus, Ki67 expression was considered positive. The number of Ki67-positive cells per 100 tumour cells was counted under a microscope using a high-power field, and the percentage was expressed as the Ki67 expression intensity. After randomly selecting ten fields of view, the average Ki67 intensity was calculated.

All patients in the study were followed up, and their recurrence, metastasis and death were recorded. The follow-up period continued through June 30, 2016.

SPSS 20.0 (IBM Corp, Armonk, NY, USA) was used for statistical analysis. Quantitative data are presented as the mean ± SD and were compared by a *t* test. Pearson linear correlation was used to assess the correlations. Receiver operating characteristic (ROC) curves were used to determine the optimal cut-off values of the Ki67 expression intensity. Recurrence-free survival (RFS) and overall survival (OS) curves were plotted using the Kaplan-Meier method and were compared with the log-rank test. *P* < 0.05 indicated significant differences.

## Results

Table 1 shows a comparison of the Ki67 expression intensity between the PTC group and the benign thyroid disease group, which included cases of nodular goiter and thyroid adenoma. The *t* test showed that the expression intensity of Ki67 within the PTC group was significantly higher than that of the benign thyroid disease group (*P* < 0.001) and that the Ki67 expression intensity of the thyroid adenoma subgroup was higher than that of the nodular goiter subgroup (*P* < 0.001).

**Table 1:** Ki67 expression intensity comparison between the benign thyroid disease group and PTC group

Type	Subtype	Case	Expression intensity (%)	*P* value
PTC		571	3.21 ± 3.466	< 0.001
Benign		228	1.63 ± 1.089	
	Nodular goiter	180	1.50 ± 1.028	< 0.001
	Thyroid adenoma	48	2.13 ± 1.178	

Table 2 summarizes the clinical and pathologic characteristics of the PTC patients. The PTC group included 434 women (76.0%) and 137 men (24.0%), with a mean age of 47.13 ± 13.08 years. The mean tumour size in the PTC group was 1.07 ± 0.86 cm. Among these patients, 159 (27.8%) had PTC with an extrathyroidal extension. Multifocality was observed in 172 (30.1%) patients, and thyroiditis was identified in 99 (17.3%) patients. CLNMs were observed in 213 (37.3%) patients.

**Table 2:** Comparison of the clinical and pathologic characteristics in PTC (*n* = 571)

Clinical characteristic		Case (*n*)	Expression intensity (%)	*P* value
Gender	Male	137	3.03 ± 3.274	0.484
	Female	434	3.27 ± 3.526	
Age	< 45 years	236	3.29 ± 3.341	0.652
	≥ 45 years	335	3.16 ± 3.555	
TGAb	≤ 115 IU/mL	456	3.00 ± 3.373	0.005
	> 115 IU/mL	115	4.03 ± 3.717	
TPOAb	≤ 34 IU/mL	476	3.09 ± 3.477	0.042
	> 34 IU/mL	95	3.91 ± 3.362	
TSH	≤ 4 uIU/ml	456	3.16 ± 3.399	0.511
	> 4 uIU/ml	115	3.40 ± 3.767	
Tumour size	< 1 cm	330	2.93 ± 3.034	0.032
	≥ 1 cm	241	3.59 ± 3.957	
Multifocality	Yes	172	3.53 ± 3.603	0.133
	No	399	3.06 ± 3.379	
Extrathyroidal extension	Yes	159	3.90 ± 1.977	0.653
	No	412	3.56 ± 3.186	
Lymph node metastasis	Yes	213	3.54 ± 4.124	0.075
	No	358	3.01 ± 2.996	
Thyroiditis	Yes	99	4.21 ± 3.942	0.005
	No	471	2.99 ± 3.322	

In the PTC group, the clinical and pathologic characteristics were compared according to the Ki67 expression intensity. Table 2 demonstrates that the expression intensity of Ki67 in a tumour greater than or equal to 1 cm in size was statistically higher than that in a tumour less than l cm in size (*P* = 0.032). The Ki67 expression intensity of the group in which thyroiditis was also present was statistically higher than that of the no thyroiditis group (*P* = 0.005). The Ki67 expression intensities in groups with high TGAb and TPOAb levels were statistically higher than those in the corresponding groups with normal levels (*P* = 0.005 and *P* = 0.042, respectively). Significant differences between the PTC group and the benign thyroid disease group in the remaining clinical and pathologic characteristics were not observed.

**Fig. 1:**
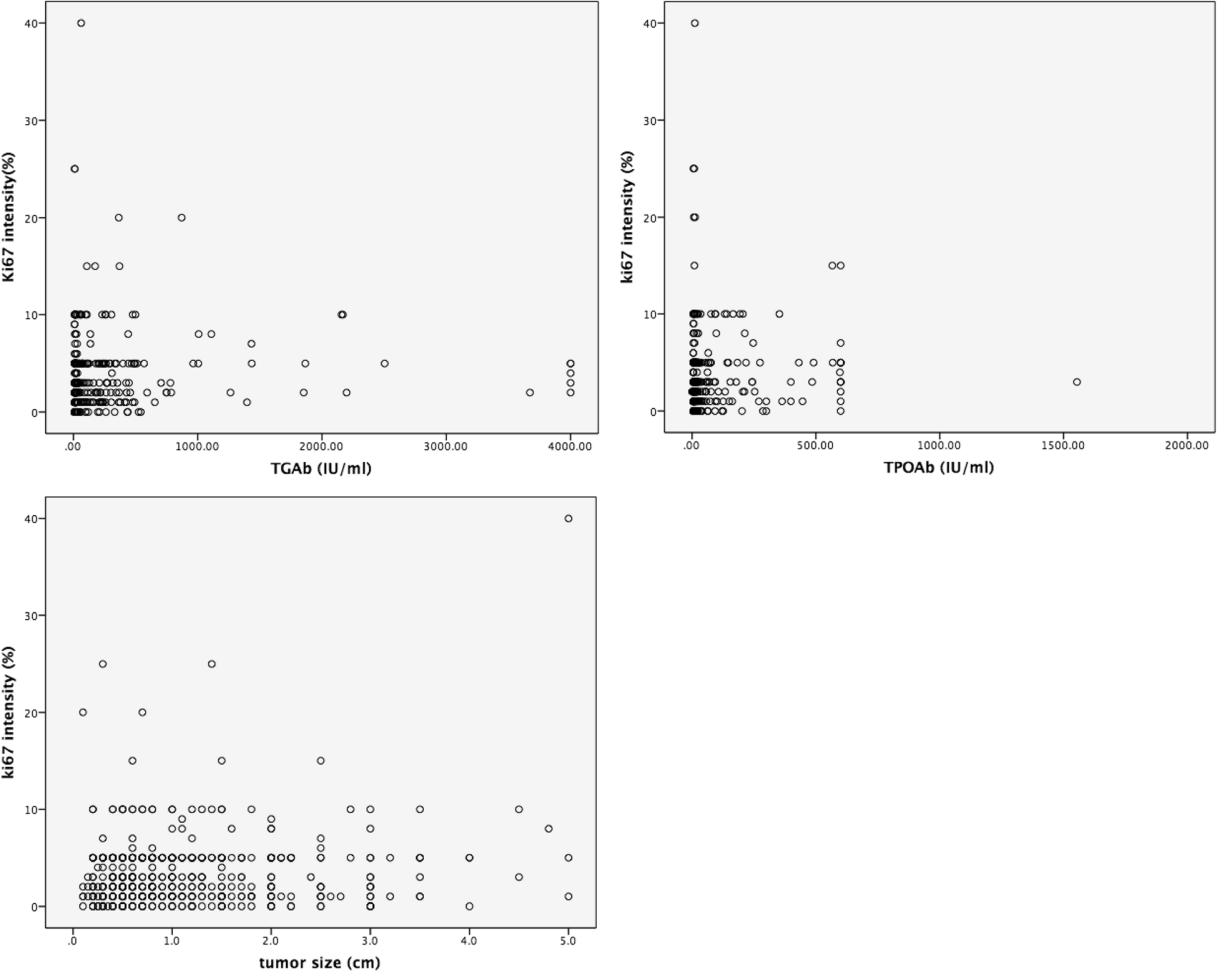
Pearson linear correlation between the Ki67 expression intensity and TGAb and TPOAb levels and tumour size showed that the TGAb and TPOAb levels had a linear correlation with the Ki67 expression intensity (*P* = 0.031 and *P* = 0.044, respectively). Tumour size also had a linear correlation with the Ki67 expression intensity (*P* < 0.001)

Table 3 and Fig. 1 show that the TGAb and TPOAb levels were linearly correlated with the Ki67 expression intensity (*P* = 0.031 and *P* = 0.044, respectively); tumour size also showed a linear correlation with the Ki67 expression intensity (*P* < 0.001).

**Table 3:** Pearson linear correlation with the Ki67 expression intensity

Characteristic	Pearson correlation	*P* value
TGAb (IU/ml)	0.090	0.031
TPOAb (IU/ml)	0.084	0.044
T3 (nmol/L)	0.008	0.837
T4 (nmol/L)	0.014	0.733
TSH (uIU/ml)	0.041	0.333
Tumour size (cm)	0.153	< 0.001
Age	0.025	0.547

**Fig. 2:**
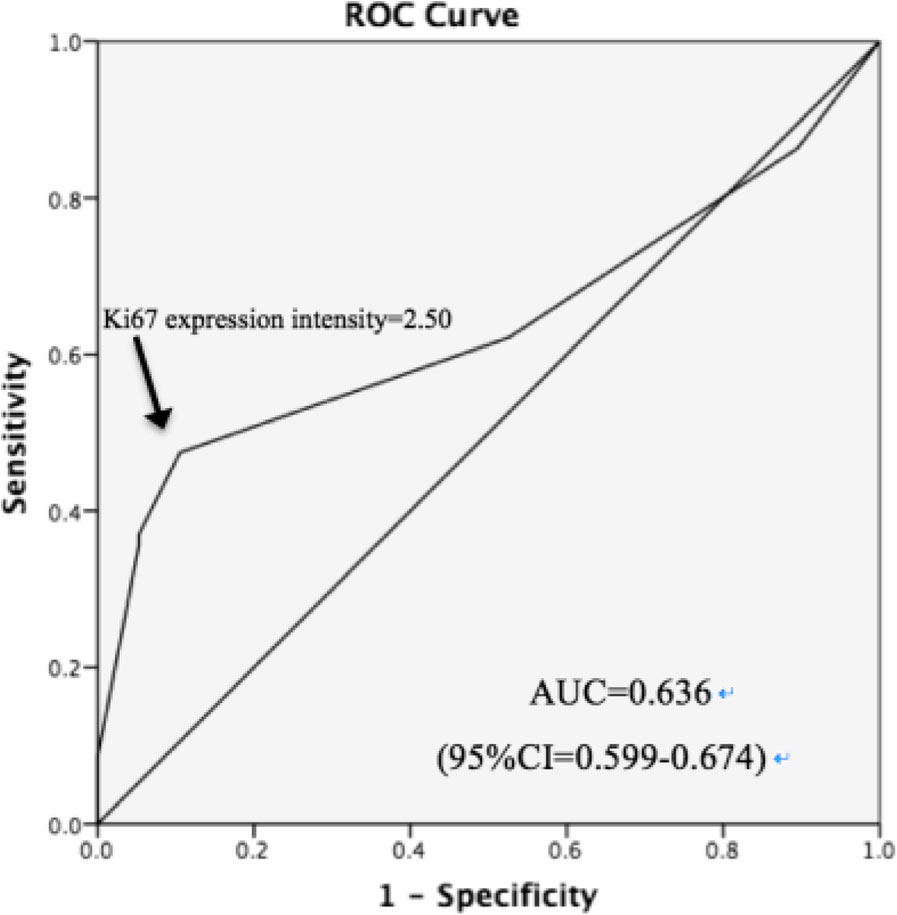
Receiver operating characteristic (ROC) curves and the cut-off value for predicting PTC, cut-off value = 2.50%, area under the curve [AUC] = 0.636 and 95% CI = 0.599–0.674

Receiver operating characteristic (ROC) curves were generated to determine the cut-off value of the Ki67 expression for the PTC cases (Fig. 2). This study suggested that the optimal cut-off value of the Ki67 expression for PTC was 2.50% (area under the curve [AUC] = 0.636, 95% CI = 0.599–0.674). As 0.5 < AUC < 0.7, Ki67 only presented a moderate diagnostic value.

Then, based on the cut-off value of the Ki67 expression intensity, patients were classified into the following two groups: group 1 (Ki67 expression intensity < 2.5%, *n* = 300) and group 2 (Ki67 expression intensity ≥ 2.5%, *n* = 271). The T stage, clinical-pathological staging, recurrence rate and mortality of the two groups were compared (Table 4). The results showed significant differences in the T stage, clinical-pathological staging and recurrence rate between the two groups.

**Table 4:** Clinical and pathologic characteristics between the two groups according to the Ki67 expression intensity

Characteristic		Group 1 (Ki67 < 2.5%) *n* = 300	Group 2 (Ki67 ≥ 2.5%) *n* = 271	*P* value
T Stage	T1	191	108	< 0.001
	T2	48	59	
	T3	62	99	
	T4	0	5	
TNM Staging	Stage I	172	111	< 0.001
	Stage II	23	36	
	Stage III	105	119	
	Stage IV	0	5	
Recurrence	Yes	1	6	0.042
	No	299	265	
Mortality	Yes	0	1	0.293
	No	300	270	

**Fig. 3:**
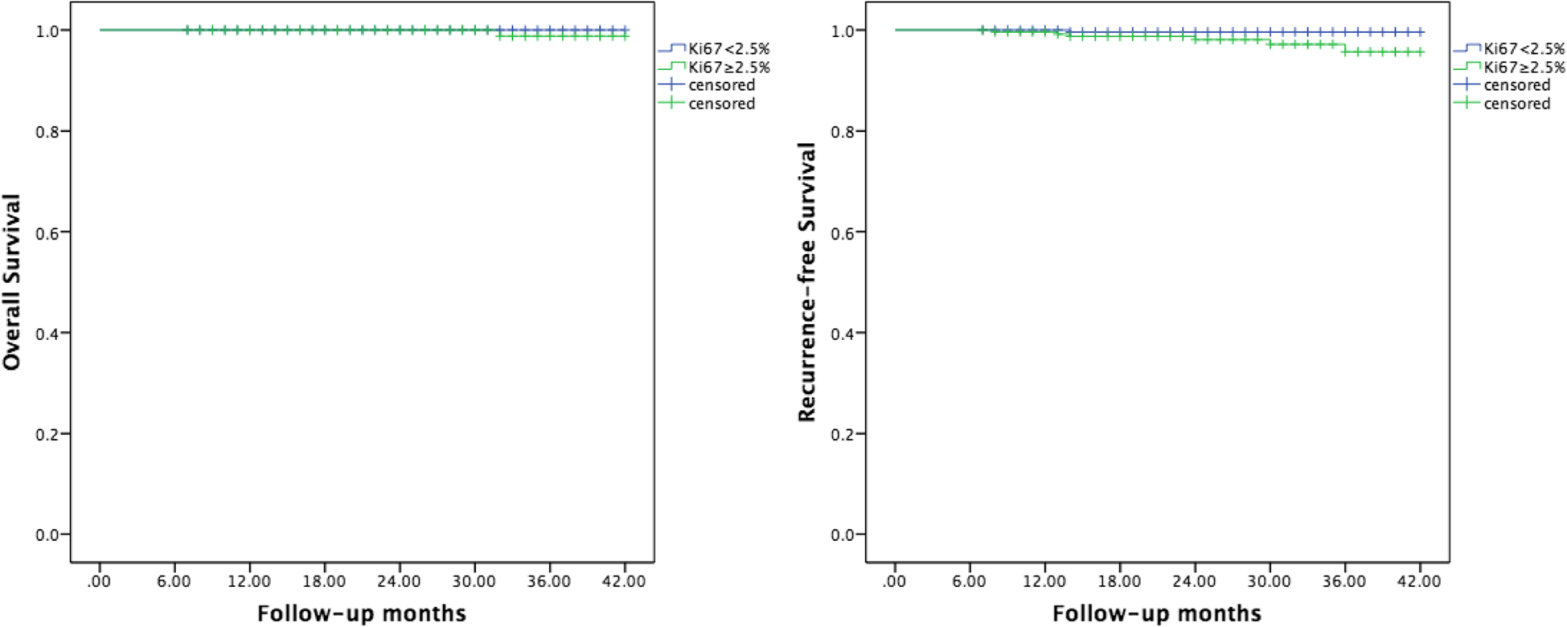
Kaplan-Meier curves of overall survival and recurrence-free survival in group 1 and group 2 according to the Ki67 expression intensity. The OS rates for patients were 100 and 99.6%, respectively (log-rank chi-square = 1.022, *P* = 0.312). The RFS of the patients were 99.7 and 97.8%, respectively (log-rank chi-square = 3.879, *P* = 0.049), which were different between the two groups

The follow-up period was, on average, 25.4 months, with the longest follow-up of 42 months and the shortest of 6 months. Seven patients had cervical lymph node metastases. One patient died of cardiovascular disease. The RFS and OS curves of group 1 and group 2 are plotted in Fig. 3, and the survival differences between the two groups were compared. The OS rates for patients were 100 and 99.6%, respectively (log-rank chi-square = 1.022, *P* = 0.312). The RFS of patients were 99.7 and 97.8%, respectively (log-rank chi-square = 3.879, *P* = 0.049), which indicated a difference between the two groups.

## Discussion

Accounting for 80–85% of thyroid cancers, PTC is the most common subtype of thyroid cancer and has increased rapidly in recent years [19, 20]. The preoperative diagnostic system for identifying benign or malignant thyroid nodules by ultrasound combined with fine-needle aspiration biopsy has been established for many years [21]. Although this approach is considered to have relatively high accuracy, it still fails to differentiate disease in some cases. Despite many efforts to improve the diagnosis and prognosis of thyroid cancer in recent years, most approaches have low specificity [22, 23]. Therefore, it is necessary to find reliable biomarkers for identifying and predicting thyroid cancer.

Ki67 is a DNA-binding protein that is mainly distributed in the nucleus and is related to cell proliferation. Ki67 is a large protein of 395 kDa, encoded by nearly 30,000 base pairs. As one of the important markers in cell proliferation, Ki67 has been widely used in the treatment and research of various types of tumours. Ki67 is usually distributed in the nucleus, and its main role is to maintain the DNA structure during cell mitosis. The primary structure of Ki67 is now known. The Ki67 protein undergoes phosphorylation and dephosphorylation during mitosis, while it is also sensitive to proteases and regulated by proteolytic pathways [24]. Moreover, the structure of Ki67 is similar to that of some proteins involved in cell cycle regulation [25]. Ki67 is absent in quiescent cells (G0) [26], but in the G1 phase, it begins to appear in the nucleus. Then, in the S and G2 phases, Ki67 protein expression gradually increases and peaks during the M phase, followed by a rapid decline during the late M phase [27].

The ki67 expression is closely related to tumour cell proliferation and growth and is widely used in routine pathology studies as a proliferation marker. It has been widely recognized that high Ki67 expression is associated with poor prognosis in breast cancer and prostate cancer [28, 29]. However, only a limited number of studies have examined Ki67 in thyroid cancer and disease. Ito et al. evaluated the prognostic significance of a Ki67 labelling index in PTC and showed that Ki67 was an independent prognostic factor for disease-free survival in PTC patients [30].

The present study revealed that the Ki67 expression intensity in the PTC group was significantly higher than that in the benign thyroid disease group and that the Ki67 expression intensity in the thyroid adenoma subgroup was higher than that in the nodular goiter subgroup. By conducting ROC analysis, we determined the optimal cut-off value of the Ki67 expression for predicting PTC to be 2.50%; however, the area under the curve was 0.636, which indicated that Ki67 expression had a relatively low diagnostic value. Therefore, Ki67 cannot be used as a single predictive factor for PTC. To improve disease prediction, some combination of factors must be used. Previous studies have suggested that evaluating TERT promoter mutations, the BRAF^V600E^ mutation or the thyroglobulin (Tg)-doubling time (DT) may be good strategies to predict PTC prognosis [31–34]. Our recent research also showed that the matrix protein periostin might be an ideal biomarker for predicting PTC prognosis [35]. In the future, Ki67 combined with another biomarker should be tested.

Our study showed that the Ki67 expression intensity was related to the following aspects of PTC, including the tumour size, coexistence with thyroiditis and pre-operative levels of TGAb and TPOAb. However, the Ki67 expression intensity was not associated with gender, age, pre-operative TSH level, multifocality, extrathyroidal extension or lymph node metastasis. This result was not exactly consistent with the previous study by Yuan Zhou et al., which showed that Ki67 expression was related to extrathyroidal extension and lymph node metastasis [36]. The reason for this inconsistency may be due to the differences in the sample size, disease spectrum and follow-up system. In our study, we only found that the Ki67 expression intensity in tumours greater than or equal to 1 cm in size was statistically higher than that in tumours less than l cm in size and that tumour size was linearly correlated with the Ki67 expression intensity. The explanation for this phenomenon could be diversity. Ki67 is a proliferation biomarker and is highly expressed in large size PTC, which includes more cells in the mitosis phase compared with smaller tumours. Therefore, a large tumour size is associated with more aggressive PTC. In fact, as the tumour size increased, the prognosis for PTC became poorer. In addition, another study reported a linear relationship between tumour size and recurrence or cancer-specific mortality in both papillary and follicular carcinomas [37, 38].

The most interesting consequence that we observed was that the expression intensity of Ki67 in cases with thyroiditis was higher than that in the no thyroiditis group. Moreover, the TGAb and TPOAb levels were linearly correlated with the Ki67 expression intensity. Thus, the Ki67 expression is highly related to thyroiditis. There are very few reports about the relationship between Ki67 expression and inflammation. Siggelkow et al. reported that Ki67 was significantly positive, with signs of inflammation in patients with silicone breast implants [39]. Edamatsu et al. stated that epithelial components of dental follicles with marked inflammatory changes showed higher rates of Ki67 expression than those without marked inflammation [40]. For thyroid disease, the relationship between Ki67 and inflammation has been reported, although the mechanism is still unknown. A study on the molecular mechanisms of Ki67 revealed that Ki67 plays an important role in the early steps of ribosomal RNA synthesis and that the Ki67 protein may be related to various signal pathways [41], which may partially answer the question of why Ki67 is associated with inflammation.

The prognosis was generally good in our research. We defined the cut-off value of the Ki67 expression and used this value to compare PTC patient prognosis. The results demonstrated a significant difference in the RFS rates between the two groups. One patient with Ki67 expression < 2.5% suffered a recurrence, and six cases in the Ki67 ≥ 2.5% group had LNM during the follow-up period. Kaplan-Meier analysis suggested that patients with higher Ki67 expression were more likely to experience disease recurrence. Therefore, this group of patients requires close follow-up with early detection and treatment.

In the present study, the diagnostic and prognostic values of Ki67 in PTC were evaluated. A Ki67 expression intensity = 2.5% was recognized as the cut-off value to predict PTC, although Ki67 needs to be combined with another molecular marker to improve its predictive effect. In addition, a relationship between thyroiditis and Ki67 expression was revealed for the first time. Nonetheless, we acknowledge the limitations of our investigation. Our study was retrospective. The follow-up time was short, and only seven recurrence incidents occurred. To confirm the conclusion, a long-term follow-up study is needed in the future.

## Conclusion

Our study verified that Ki67 is highly expressed in papillary thyroid carcinoma and can be used to differentiate PTC from benign thyroid disease. The expression intensity of Ki67 in PTC is related to the tumour size, thyroiditis and TGAb and TPOAb levels. PTC patients with highly expressed Ki67 are more likely to experience disease recurrence. Ki67 could be an important indicator for judging tumour aggressiveness and inflammatory lesions.
